# Comparison of core temperature using tracheal thermometer and pulmonary artery catheter in adult patients undergoing coronary artery bypass graft surgery

**DOI:** 10.1371/journal.pone.0314322

**Published:** 2025-01-02

**Authors:** Seyeon Park, Hee Young Kim, Hye-Jin Kim, Jieun Jung, Seo-Ho Hong, Yeon-Soo Jung, Dong-Hyeon Ha, Da-Eun Park, Ji-Uk Yoon

**Affiliations:** 1 Department of Anesthesia and Pain Medicine, Pusan National University Yangsan Hospital, Yangsan, Korea; 2 Department of Anesthesia and Pain Medicine, School of Medicine, Pusan National University, Yangsan, Republic of Korea; 3 School of Medicine, Pusan National University, Yangsan, Republic of Korea; Ataturk University Faculty of Medicine, TÜRKIYE

## Abstract

**Background:**

Monitoring core temperature is important for patients under anesthesia. Esophageal and pulmonary artery blood temperatures can be used for measuring core temperature during general anesthesia. However, these methods pose challenges, especially when the placement of an esophageal thermometer and pulmonary artery catheter (PAC) is either impractical or not the preferred approach. An endotracheal tube (ETT) with a thermometer on the cuff allows for the measurement of tracheal temperature, providing a suitable alternative to core temperature measurement. This study aimed to assess the clinical reliability and accuracy of the thermometer in the ETT in comparison to the core temperature measured using a PAC.

**Methods:**

Eleven patients who underwent coronary artery bypass graft (CABG) surgery were enrolled in this study. The patients were intubated using an ETT equipped with a thermometer on the cuff, and a PAC was inserted. Temperature measurements of both the trachea and pulmonary artery blood were recorded at 5-minute intervals for 1 hour before starting cardiopulmonary bypass. The agreement between the two temperature measurement methods was investigated using the Bland-Altman plot with multiple measurements per subject, and the correlation was evaluated using the concordance correlation coefficient (CCC).

**Results:**

Eleven patients with a total of 143 pairs of data were included for analysis. The mean difference between the tracheal and pulmonary artery temperatures was −0.10°C. The 95% limit of agreement (LoA), calculated as ± 1.96 standard deviation, ranged from −0.35°C to 0.15°C. The 95% confidence interval for the lower and upper LoA was −0.51°C to −0.27°C and 0.07°C to 0.31°C, respectively. The maximum allowed difference (Δ) was set at 0.5°C. The majority of temperature differences fell within the LoA and were well below the maximum allowed difference. The CCC was 0.95, which indicates a substantial strength of agreement.

**Conclusions:**

The agreement between the tracheal and pulmonary artery temperature measurements using the ETT thermometer and pulmonary artery catheter, respectively, was found to be clinically reliable and accurate. Therefore, the tracheal temperature measurement can effectively represent the core temperature of the patients. Employing an ETT equipped with a thermometer on the cuff can serve as a reliable and independent method for measuring core temperature.

**Trial registration:**

**Clinical trial registration number:** NCT05595616.

## Introduction

Monitoring core temperature is important for patients under anesthesia. Temperature control and monitoring are critical, especially in cases of hypothermia or malignant hyperthermia in the operating room [1, 2]. Core temperature can be measured at various sites, including the pulmonary artery, esophagus, tympanic membrane, and nasopharynx. The esophagus is a commonly used site for temperature measurement in patients under general anesthesia [1, 2]. However, in cases where monitoring core temperature at the esophagus is challenging, such as in surgeries involving the head and neck or esophagus, alternative methods may need to be considered [3].

An endotracheal tube (ETT) with a built-in thermometer allows for the measurement of tracheal temperature, which can be a suitable alternative to esophageal temperature measurement [4, 5]. This method eliminates the need for a separate esophageal thermometer, providing a convenient and reliable means of monitoring core temperature during anesthesia.

The measurement of pulmonary artery blood temperature using a pulmonary artery catheter (PAC) is the gold standard for monitoring core temperature [2, 4]. In this study, we compared the tracheal temperature measured using an ETT thermometer with the blood temperature measured using a PAC. The study aimed to assess the clinical reliability and accuracy of the ETT thermometer compared to core temperature measured using a PAC.

## Materials and methods

### Participants

**Fig 1:**
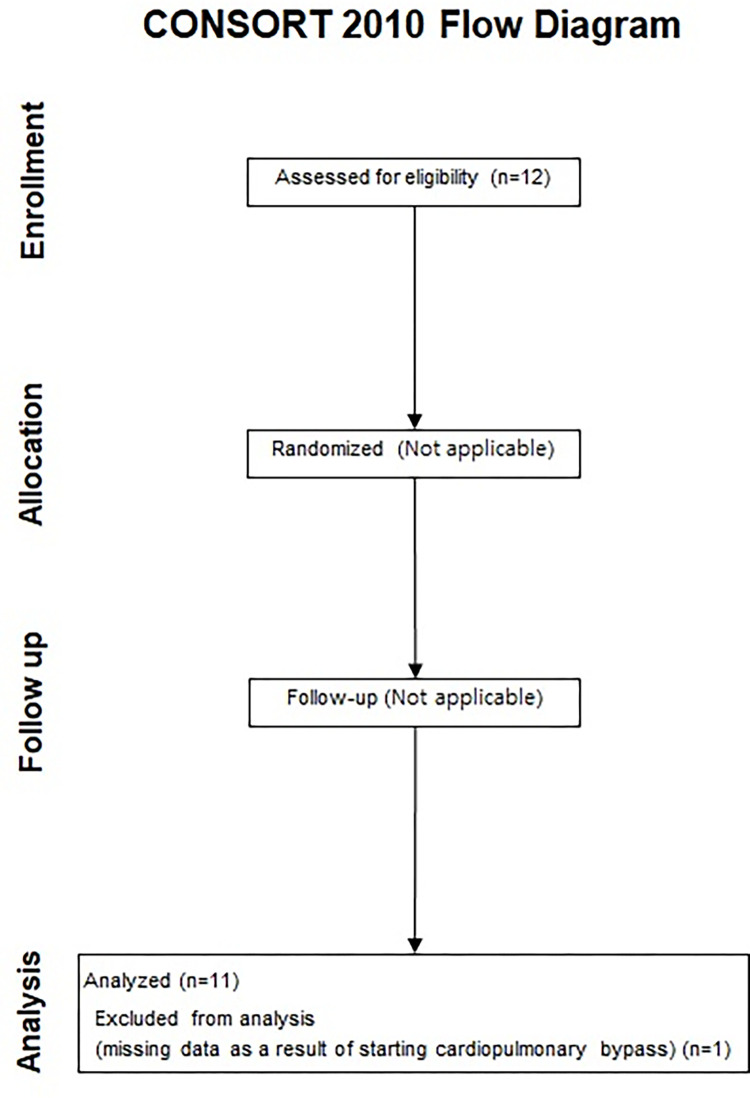
CONSORT flow diagram.

This observational cohort study was approved by the Institutional Review Board of Pusan National University Yangsan Hospital (IRB No. 05-2022-190) and registered at ClinicalTrials.gov (NCT05595616). The study was conducted in accordance with the Declaration of Helsinki (as revised in 2013). A total of 12 patients aged >18 years who underwent coronary artery bypass graft (CABG) surgery with PAC insertion were enrolled (from September 20, 2022, to November 21, 2022), and written informed consent was obtained. Exclusion criteria were patients with unstable vital signs who underwent emergency surgeries. After enrolment, patients with an inappropriate position of the PAC and thermometer malfunction were excluded from the study. The CONSORT flow diagram is presented in Fig 1.

### Anesthesia and temperature measurement

**Fig 2:**
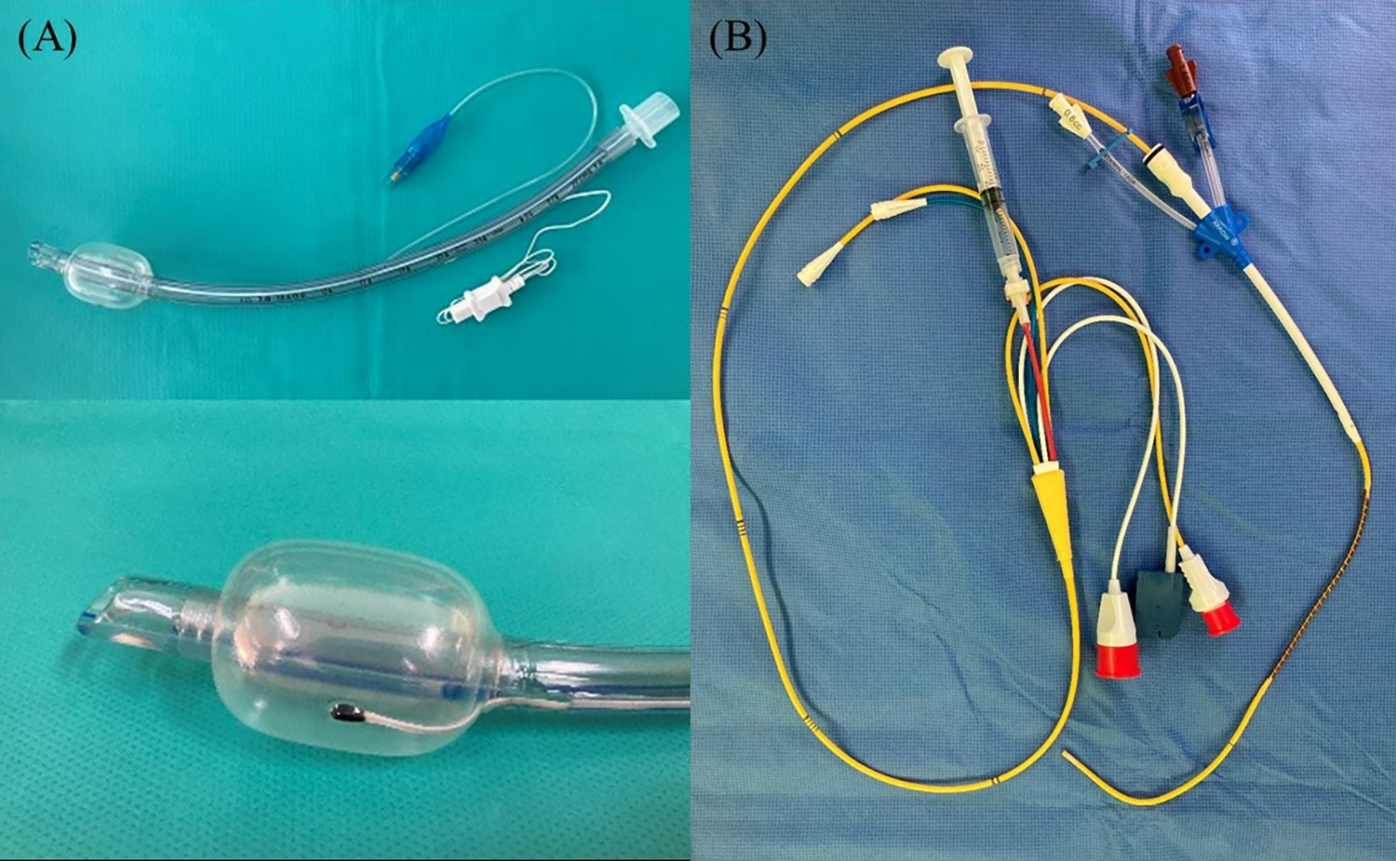
(A) Endotracheal tube equipped with a thermometer on the cuff. (B) Pulmonary artery catheter with a 9 French central catheter.

All patients underwent standard monitoring in the operating room, which included non-invasive blood pressure measurement, electrocardiogram, and pulse oximetry. After the induction of general anesthesia using 1–2 mg/kg of 1% propofol, 0.8 mg/kg of rocuronium, and remifentanil, the anesthesia was maintained with sevoflurane and an O_2_-air mixture. The patients were intubated using an ETT equipped with a thermometer on the cuff (Human Endo, Insung Medical Co., Korea; Fig 2A). Following central catheterization of the right internal jugular vein, a PAC (Swan-Ganz CCOmbo V, Edward Lifesciences, Irvine, California, USA; Fig 2B) was inserted, and its placement was confirmed using transesophageal echocardiography (TEE). Subsequently, tracheal temperature (*T*_*T*_) was measured using the ETT thermometer, and blood temperature (*T*_*P*_) was obtained using the PAC. Temperature measurements were taken at 5-minute intervals for 1 hour before starting cardiopulmonary bypass (CPB). Follow-up observations for the study are not required, and the research concludes on the same day.

### Statistical analysis and sample size

The primary outcome of the study was to determine whether the tracheal temperature, measured using the ETT thermometer, accurately reflected the core temperature measured using the PAC. We assessed the reliability and accuracy of the ETT thermometer as an alternative for core temperature monitoring and analyzed the agreement and correlation between these two temperature measurements. The agreement was investigated using the Bland–Altman plot with multiple measurements per subject, and the correlation was evaluated using the concordance correlation coefficient (CCC) [6].

The sample size was calculated based on methods used in previous studies [7, 8]. We assumed that the mean difference between the tracheal and core temperatures was approximately 0.25°C, the standard deviation of the difference was approximately 0.1°C, and the maximum allowed difference between methods was 0.5°C [2]. Assuming a two-side *α* of 0.05, a *β* of 0.1 (power of 0.9), the minimum required number of pairs was calculated to be 111. Since 13 pairs of data were collected per participant (data collected every 5 minutes for 1 hour), the result of 111 pairs of data divided by 13 was approximately 9 (8.5) participants. Considering the attrition rate of 20%, 12 patients were recruited.

All data are expressed as numbers (proportions), the mean ± standard deviation, or median (interquartile range), unless otherwise specified. Statistical analyses were performed using MedCalc^®^ Statistical Software, version 22.007 (MedCalc software Ltd., Ostend, Belgium).

## Results

A total of 12 patients were enrolled from September 20, 2022, to November 21, 2022. However, one patient was later excluded due to missing data as a result of starting CPB. Therefore, 11 patients were finally enrolled, with a total of 143 pairs of data included for analysis. The patient characteristics are summarized in Table 1, and all measured data are presented in S1 Table.

**Table 1:** Patient characteristics.

	n = 11
Age (year)	64 (60–72) [55–76]
Male	9
Female	2
Height (cm)	165.7 ± 9.2
Weight (kg)	70.0 ± 4.0

Values are presented as the mean ± standard deviation, median (interquartile range) [range], or number.

**Fig 3:**
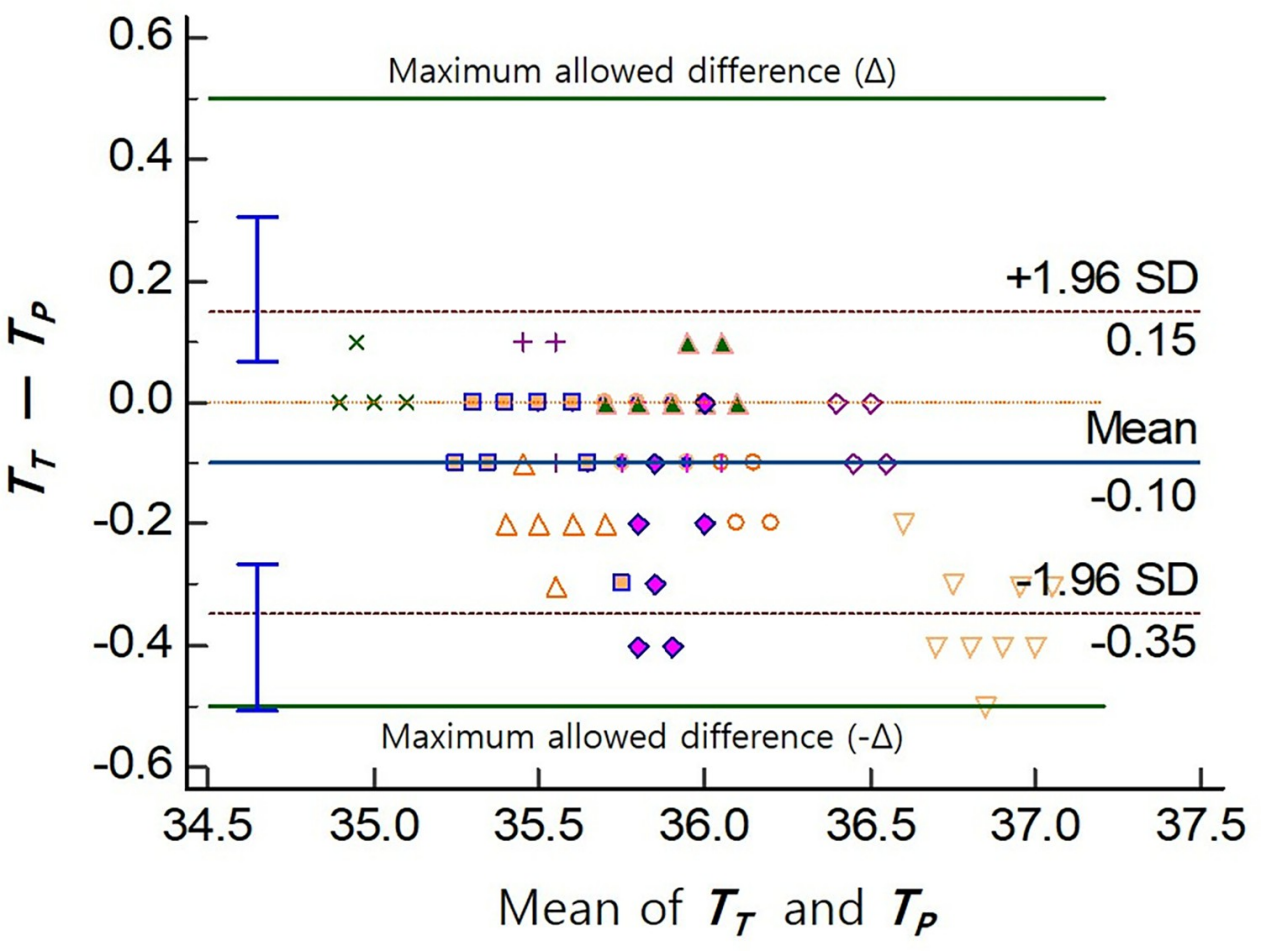
Bland–Altman plot. The mean difference between the tracheal and pulmonary artery temperatures was −0.10°C. The 95% limit of agreement (LoA), calculated as ± 1.96 standard deviations, ranged from −0.35°C to 0.15°C. The 95% confidence interval for the lower and upper LoA was −0.51°C to −0.27°C and 0.07°C to 0.31°C, respectively. The maximum allowed difference (Δ) was set at 0.5°C. The majority of temperature differences fell within the LoA and were well below the maximum allowed difference, suggesting a good agreement between the two measurement methods. *T*_*T*_, tracheal temperature; *T*_*P*_, pulmonary artery temperature.

**Fig 4:**
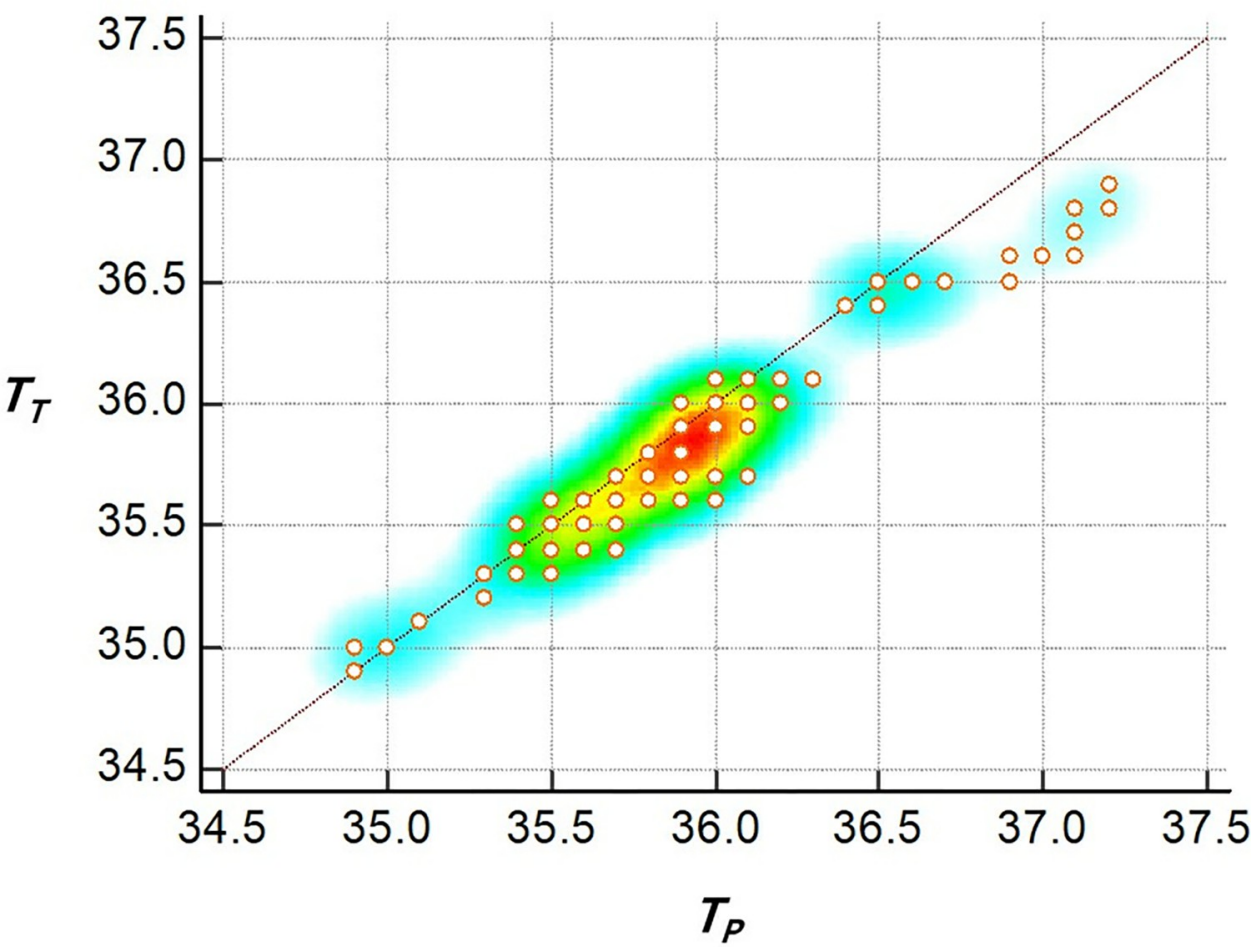
Scatter diagram for concordance correlation coefficient. The concordance correlation coefficient was 0.95, indicating a substantial strength of agreement. *T*_*T*_, tracheal temperature; *T*_*P*_, pulmonary artery temperature; Red colors, high incidence levels; Blue colors, low incidence levels.

The primary outcome, which was the agreement between the tracheal and pulmonary artery temperature measurements using the ETT thermometer and PAC, respectively, was found to be significant. The mean difference between the tracheal and pulmonary artery temperatures was −0.10°C. The 95% limit of agreement (LoA), calculated as ± 1.96 standard deviation, ranged from −0.35°C to 0.15°C. The 95% confidence interval for the lower and upper LoA was −0.51°C to −0.27°C and 0.07°C to 0.31°C, respectively (Table 2). These values indicate the range within which most temperature differences between the two methods are included. Additionally, the maximum allowed difference (Δ) was set at 0.5°C. The majority of temperature differences fell within the LoA and were well below the maximum allowed difference, suggesting a good agreement between the two measurement methods (Fig 3). Furthermore, the CCC was 0.95, indicating a substantial strength of agreement (Fig 4).

**Table 2:** The differences between tracheal and pulmonary artery temperatures.

	*T*_*T*_ vs. *T*_*P*_
Mean difference,°C	-0.10
Lower limit of agreement,°C	-0.35
(95% confidence interval)	(-0.51 to -0.27)
Upper limit of agreement,°C	0.15
(95% confidence interval)	(0.07 to 0.31)
Concordance correlation coefficient	0.95

The agreement between the tracheal and pulmonary artery temperature measurements using the endotracheal tube thermometer and pulmonary artery catheter, respectively, was found to be significant. The mean difference between the tracheal and pulmonary artery temperatures was −0.10°C. The 95% limit of agreement (LoA), calculated as ± 1.96 standard deviation, ranged from −0.35°C to 0.15°C. The 95% confidence interval for the lower and upper LoA was −0.51°C to -0.27°C and 0.07°C to 0.31°C, respectively. The concordance correlation coefficient was 0.95, indicating a substantial strength of agreement. *T*_*T*_, tracheal temperature; *T*_*P*_, pulmonary temperature.

## Discussion

In this study, the agreement between the tracheal and pulmonary artery temperature measurements using the ETT thermometer and PAC, respectively, was found to be clinically reliable and accurate, which indicates that the tracheal temperature measurement can effectively represent the core temperature of the patients. Consequently, the use of an ETT equipped with a thermometer on the cuff can be considered a viable alternative for measuring core temperature. This method offers practicality and accuracy, particularly in situations where the placement of a PAC and esophageal thermometer may not be feasible or preferred.

Another study involving patients who underwent living donor liver transplantation reported similar results to our study, demonstrating the reliability of tracheal temperature monitoring with a percentage error of −0.15% [9]. In our study, the percentage error was −0.28%. A low percentage error suggests that the tracheal temperature monitoring method provides accurate and precise measurements.

The ETT used in our study had a thermometer on the cuff; therefore, temperature measurements were less affected by a breathing circuit with a heated wire humidifier [4, 10]. The ETT cuff pressure (via a manometer) for all participants was consistent. No complications due to the cuff thermometer were observed.

The pulmonary artery temperature measurement is less commonly used than esophageal temperature monitoring, which is the usual method used in patients under general anesthesia [2, 4]. However, there are certain situations where esophageal temperature measurement may not be applicable or feasible. These include patients with esophageal diseases, such as severe varices, as well as surgeries involving the head and neck, thorax, or esophagus [2]. In particular, the use of TEE during cardiac and transplant surgeries can pose challenges for core temperature monitoring using esophageal temperature probes. The TEE probe can interfere with the proper positioning and functioning of esophageal temperature probes, leading to inaccurate temperature measurements [2]. In these cases, alternative methods for monitoring core temperature need to be considered. Tracheal temperature monitoring can serve as a reliable and independent method for measuring core temperature.

Nevertheless, our study has several limitations. This study was conducted at a single center, and the sample size was relatively small. However, other studies have reported similar results, supporting the findings of our study [4, 5, 8, 9, 11]. This consistency in the literature enhances the overall reliability and validity of the results, despite the limitations.

In conclusion, the agreement between the tracheal and pulmonary artery temperature measurements using the ETT thermometer and PAC, respectively, was found to be clinically reliable and accurate. These findings indicate that the tracheal temperature measurement can effectively represent the core temperature of the patients. The use of an ETT equipped with a thermometer on the cuff can be a reliable and independent method for measuring core temperature.

## Supporting information

S1 Table Data set of enrolled participants. (DOCX)

## References

[1] Evron S, Weissman A, Toivis V, Shahaf DB, You J, Sessler DI, et al. Evaluation of the temple touch pro, a novel noninvasive core-temperature monitoring system. Anesthesia & Analgesia. 2017;125(1):103–9. 28617697 10.1213/ANE.0000000000001695

[2] Sessler DI. Perioperative Temperature Monitoring. Anesthesiology. 2021;134(1):111–8. doi: 10.1097/ALN.0000000000003481 32773677

[3] Hymczak H, Gołąb A, Mendrala K, Plicner D, Darocha T, Podsiadło P, et al. Core Temperature Measurement—Principles of Correct Measurement, Problems, and Complications. International Journal of Environmental Research and Public Health. 2021;18(20):10606. doi: 10.3390/ijerph182010606 34682351 PMC8535559

[4] Hayes J, Collette D, Smith K, Peters J. Monitoring Body Core Temperature from the Trachea. ANESTHESIOLOGY-PHILADELPHIA THEN HAGERSTOWN-. 1995;83:A402-A.

[5] Kawano Y, Imai M, Komura Y, Kikuchi N, Kemmotsu O. Tracheal cuff as a new core temperature site. Anesthesiology. 1992;77:A563.

[6] Bland JM, Altman DG. Statistical methods for assessing agreement between two methods of clinical measurement. International journal of nursing studies. 2010;47(8):931–6.

[7] Lu M-J, Zhong W-H, Liu Y-X, Miao H-Z, Li Y-C, Ji M-H. Sample size for assessing agreement between two methods of measurement by Bland− Altman method. The International Journal of Biostatistics. 2016;12(2). doi: 10.1515/ijb-2015-0039 27838682

[8] Haugk M, Stratil P, Sterz F, Krizanac D, Testori C, Uray T, et al. Temperature monitored on the cuff surface of an endotracheal tube reflects body temperature. Critical care medicine. 2010;38(7):1569–73. doi: 10.1097/CCM.0b013e3181e47a20 20495450

[9] Yang S-M, Cho H-Y, Kim H-S. Comparison of tracheal temperature and core temperature measurement in living donor liver transplant recipients: a clinical comparative study. BMC anesthesiology. 2022;22(1):1–7. 36217113 10.1186/s12871-022-01853-9 PMC9549662

[10] Thomachot L, Viviand X, Lagier P, Marc Dejode J, Albanèse J, Martin C. Measurement of tracheal temperature is not a reliable index of total respiratory heat loss in mechanically ventilated patients. Critical Care. 2000;5(1):1–7. 10.1186/cc974 PMC29053 11178222

[11] Yamakage M, Kawana S, Watanabe H, Namiki A. The utility of tracheal temperature monitoring. Anesthesia & Analgesia. 1993;76(4):795–9. doi: 10.1213/00000539-199304000-00020 8466020

