# Supplementary material for: Comparison of core temperature using tracheal thermometer and pulmonary artery catheter in adult patients undergoing coronary artery bypass graft surgery
Source: PLoS One. 2025 Jan 2;20(1):e0314322. doi: 10.1371/journal.pone.0314322 (PMC11694998; doi:10.1371/journal.pone.0314322)
Supplement: S1 Table — (DOCX) [file pone.0314322.s001.docx]

**S1 Table. Data set of enrolled participants**

| **No.** | **1** | | **2** | | **3** | | **4** | | **5** | | **6** | | **7** | | **8** | | **9** | | **10** | | **11** | | **12** | |
| --- | --- | --- | --- | --- | --- | --- | --- | --- | --- | --- | --- | --- | --- | --- | --- | --- | --- | --- | --- | --- | --- | --- | --- | --- |
|  | ***T_T_ (℃)*** | ***T_P_ (℃)*** | ***T_T_ (℃)*** | ***T_P_ (℃)*** | ***T_T_ (℃)*** | ***T_P_ (℃)*** | ***T_T_ (℃)*** | ***T_P_ (℃)*** | ***T_T_ (℃)*** | ***T_P_ (℃)*** | ***T_T_ (℃)*** | ***T_P_ (℃)*** | ***T_T_ (℃)*** | ***T_P_ (℃)*** | ***T_T_ (℃)*** | ***T_P_ (℃)*** | ***T_T_ (℃)*** | ***T_P_ (℃)*** | ***T_T_ (℃)*** | ***T_P_ (℃)*** | ***T_T_ (℃)*** | ***T_P_ (℃)*** | ***T_T_ (℃)*** | ***T_P_ (℃)*** |
| **T_0_** | 35.9 | 36.0 | 35.6 | 35.6 | 35.9 | 36.0 | 35.1 | 35.1 | 35.9 | 36.0 | 35.5 | 35.7 | 35.6 | 35.9 | 36.1 | 36.1 | 36.0 | 36.0 | 36.5 | 36.5 | 36.5 | 36.5 | 36.5 | 36.7 |
| **T_1_** | 35.9 | 36.0 | 35.6 | 35.7 | 35.9 | 36.0 | 35.1 | 35.1 | 35.9 | 36.1 | 35.4 | 35.7 | 35.6 | 35.7 | 36.1 | 36.0 | 35.9 | 36.1 | 36.5 | 36.6 | 36.6 | 36.6 | 36.5 | 36.7 |
| **T_2_** | 36.0 | 36.0 | 35.6 | 35.6 | 35.9 | 36.0 | 35.1 | 35.1 | 36.0 | 36.1 | 35.4 | 35.6 | 35.6 | 35.6 | 36.0 | 35.9 | 35.8 | 35.9 | 36.5 | 36.6 | 36.6 | 36.6 | 36.5 | 36.9 |
| **T_3_** | 36.0 | 36.1 | 35.6 | 35.5 | 35.9 | 36.0 | 35.1 | 35.1 | 36.0 | 36.1 | 35.3 | 35.5 | 35.5 | 35.5 | 36.0 | 35.9 | 35.8 | 35.9 | 36.5 | 36.6 | 36.6 | 36.6 | 36.6 | 36.9 |
| **T_4_** | 36.0 | 36.1 | 35.5 | 35.5 | 35.9 | 35.9 | 35.0 | 34.9 | 36.0 | 36.0 | 35.4 | 35.5 | 35.4 | 35.4 | 36.0 | 36.0 | 35.8 | 35.9 | 36.5 | 36.5 | 36.6 | 36.6 | 36.6 | 37.0 |
| **T_5_** | 36.0 | 36.1 | 35.5 | 35.5 | 35.8 | 35.8 | 35.0 | 35.0 | 35.8 | 35.8 | 35.4 | 35.6 | 35.3 | 35.4 | 36.0 | 36.0 | 35.8 | 35.9 | 36.5 | 36.5 | 36.6 | 36.6 | 36.6 | 37.1 |
| **T_6_** | 36.0 | 36.1 | 35.5 | 35.5 | 35.7 | 35.8 | 35.0 | 35.0 | 35.8 | 35.8 | 35.4 | 35.5 | 35.3 | 35.4 | 36.0 | 35.9 | 35.7 | 35.9 | 36.5 | 36.5 | 36.6 | 36.6 | 36.7 | 37.1 |
| **T_7_** | 36.0 | 36.2 | 35.5 | 35.5 | 35.7 | 35.8 | 35.0 | 35.0 | 35.7 | 35.8 | 35.4 | 35.5 | 35.3 | 35.4 | 35.9 | 35.9 | 35.7 | 35.9 | 36.4 | 36.5 | 36.6 | 36.6 | 36.8 | 37.1 |
| **T_8_** | 36.0 | 36.2 | 35.5 | 35.5 | 35.7 | 35.7 | 35.0 | 35.0 | 35.7 | 35.8 | 35.4 | 35.6 | 35.3 | 35.4 | 35.9 | 35.9 | 35.6 | 36.0 | 36.4 | 36.5 | 36.6 | 36.6 | 36.8 | 37.1 |
| **T_9_** | 36.0 | 36.2 | 35.4 | 35.5 | 35.7 | 35.7 | 35.0 | 35.0 | 35.7 | 35.8 | 35.4 | 35.7 | 35.3 | 35.3 | 35.9 | 35.9 | 35.7 | 36.0 | 36.4 | 36.5 | 36.1 | 36.0 | 36.8 | 37.2 |
| **T_10_** | 36.1 | 36.2 | 35.5 | 35.6 | 35.6 | 35.6 | 34.9 | 34.9 | 35.7 | 35.8 | 35.6 | 35.8 | 35.2 | 35.3 | 35.9 | 35.9 | 35.7 | 36.0 | 36.4 | 36.4 |  |  | 36.9 | 37.2 |
| **T_11_** | 36.1 | 36.3 | 35.5 | 35.5 | 35.6 | 35.6 | 34.9 | 34.9 | 35.7 | 35.8 | 35.6 | 35.8 | 35.2 | 35.3 | 35.8 | 35.8 | 35.7 | 36.0 | 36.4 | 36.5 |  |  | 36.9 | 37.2 |
| **T_12_** | 36.1 | 36.3 | 35.5 | 35.4 | 35.6 | 35.6 | 34.9 | 34.9 | 35.7 | 35.8 | 35.6 | 35.8 | 35.2 | 35.3 | 35.7 | 35.7 | 35.7 | 36.1 | 36.4 | 36.5 |  |  | 36.9 | 37.2 |

No., Participant number; T_number_, Time point of temperature measurements, *T_T_*, tracheal temperature; *T_P_*, pulmonary temperature.

After enrolment, patient No.11 was excluded later due to missing data resulting from the initiation of cardiopulmonary bypass.
